# Distribution and association of cancer with mortality in end-stage renal disease patients receiving dialysis

**DOI:** 10.1007/s40620-019-00649-4

**Published:** 2019-09-25

**Authors:** Rajkumar Chinnadurai, Emma Flanagan, Philip A. Kalra

**Affiliations:** 1 Department of Renal Medicine, Salford Royal NHS Foundation Trust, Salford, M6 8HD UK; 2 Faculty of Biology, Medicine and Health, University of Manchester, Manchester, UK; 3 Information Management and Technology, Salford Royal NHS Foundation Trust, Salford, UK

**Keywords:** Cancer, End-stage renal disease, Dialysis, All-cause mortality, Onconephrology

## Abstract

**Background and aims:**

Cancer in end-stage renal disease (ESRD) patients is an important comorbidity to be taken into consideration while planning for renal replacement therapy (RRT) options due to its associated increased mortality. This study aims to investigate the natural history and association of cancer with all-cause mortality in an ESRD population receiving dialysis.

**Method:**

The study was conducted on 1271 ESRD patients receiving dialysis between January 2012 and December 2017. A comparative analysis was carried out between 119 patients with and 1152 without cancer history at entry into this study (baseline). A 1:2 (119 cancer: 238 no cancer) propensity score matched sample of 357 patients was also used for analysis. Cox-regression analysis was used to study the strength of the association between cancer and all-cause mortality. Kaplan–Meier (KM) analysis was used to demonstrate the difference in cumulative survival between the groups. A competing risk analysis was also carried out to calculate the probability of competing events (death, transplant and incident cancer).

**Results:**

At baseline, 10.1% of the cohort had a history of cancer (current and past) with the annual incident rate being 1.3%. Urological cancers were the leading site of cancer. The median age of our cohort was 63 years with a predominance of males (63%) and Caucasians (79%). The majority (69%) of the cohort were receiving haemodialysis. 47% had a history of diabetes with 88% being hypertensive. During a median follow-up of 28 months, the proportion of deaths observed was similar between the groups in the matched sample (cancer 49.6 versus no-cancer 52.1%, *p* value 0.77). In a univariable Cox-regression model, there was no significant association between cancer and all-cause mortality (HR 1.28; 95% CI 0.97–1.67; *p* = 0.07). The KM estimates showed similar observations in the cumulative survival between the groups (matched sample log-rank, *p* value 0.85). In competing risk analysis, the cumulative probability of death at 5 years was non-significantly higher in the cancer group (cancer group 64% vs no cancer group 51%, *p* value 0.16).

**Conclusions:**

In our real-world multi-morbid dialysis cohort of 119 cancer patients, baseline cancer history did not prove to be an independent risk factor for all-cause mortality in the first 5 years of follow-up, suggesting the need for a case-by-case approach in provision of RRT options, including transplantation.

## Introduction

Cancer in end-stage renal disease (ESRD) patients receiving dialysis can be an added burden to their overall morbidity and mortality. Urological cancers including cancers of kidney, prostate, bladder and urinary tract are noted to be the leading sites of cancer in ESRD patients, which is of no surprise given their anatomical situation [1]. An increased incidence of cancer in patients after kidney transplantation due to immunosuppression is well reported. However, it has become increasingly evident that there is an increased incidence of cancer even in ESRD patients on dialysis because of the effects of uraemic toxins [2, 3]. Cancer risk was not found to be different between patients receiving haemodialysis or peritoneal dialysis modalities [4]. The United States Renal Data System (USRDS) annual data from 2016 reports the prognosis of ESRD patients with cancer to be worse than those without, although cardiovascular disease is still the leading cause of death in this population [5]. A recent study from the Australian and New Zealand dialysis and transplant registry showed a > 2.5-fold increased risk of cancer death in dialysis and transplant recipients compared to the general population [6].

Examining the natural history of cancer in ESRD patients can help in the decision-making process for renal replacement therapy and in planning treatment withdrawal. Several recent studies looking at cancer and its association with outcomes in ESRD are reported in East Asian ethnic groups [7, 8]. However, such studies are scarce in a Caucasian population with patients managed in an entirely different health care system. This study aims to investigate the distribution of cancer and its association with mortality in ESRD patients receiving dialysis in a United Kingdom (UK) renal service.

## Methods

### Sampling

**Fig. 1:**
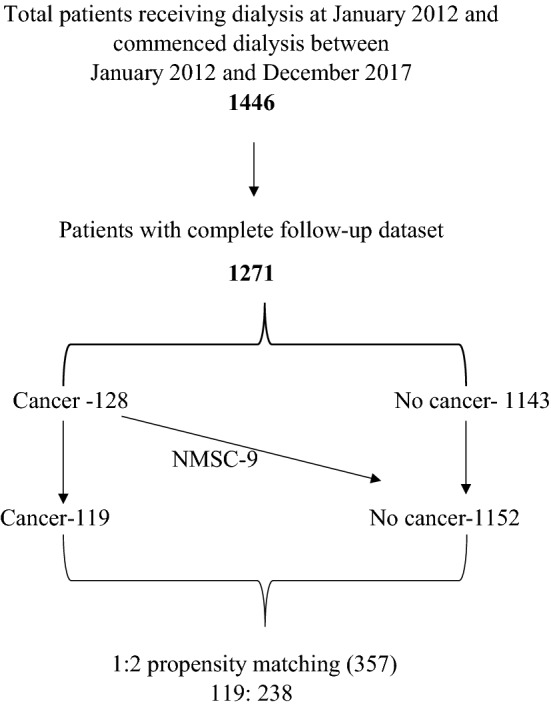
Flowchart of patient recruitment to the study

This cross-sectional observational study was conducted on ESRD patients receiving dialysis at Salford Royal Hospital (a tertiary renal centre in the UK with a catchment population of 1.55 million) and its four satellite units. A list of all patients who received chronic dialysis between 1st January 2012 and 31st December 2017 was generated from the hospital electronic patient records (EPR). From this list of 1446 patients, a sample of 1271 patients who were receiving dialysis for at least 3 months from recruitment and with complete follow-up datasets were included in this study. Of the 1271 patients, 128 had a history of cancer at this study entry (baseline). Further comparative analysis was carried out between 119 patients with cancer and 1152 patients without cancer, after including nine patients with non-melanoma skin cancer (NMSC) in the no cancer group. A 1:2 (119 cancer: 238 no cancer) propensity score matched sample of 357 patients generated from these groups was also used in the analysis. A flowchart of patient recruitment to the study is shown in Fig. 1.

### Data collection

At study baseline, data including demographics, co-morbidities, physical parameters (weight, height and blood pressure) and blood results were gathered. Follow-up data collected included incident cancer details, transplant date and date of death. The baseline study date was taken as 1/1/2012 for those patients who started dialysis before that date and the dialysis start date for the remaining of the patients. All patients were followed-up until reaching a study endpoint which included death, renal transplant, or an arbitrary end date 31/10/2018. Comprehensive cause of death data was unavailable for the patients in this cohort.

### Statistics

The 119 cancer patients were matched for age, gender and ethnicity with the 1152 patients without cancer at baseline by propensity score matching. Matching was undertaken using three major variables age, gender and ethnicity by a 1:2 neighbour match of patients with the same propensity scores that were generated by the `MatchIt’ package of the R software version 3.5.1 [9, 10]. The resulting matched cohort of 357 patients was used for comparative analysis. In the comparative analysis, the continuous variables were expressed as median (interquartile range) with the Mann–Whitney *U* test used to define the *p* value while categorical data were expressed as number (percentage) and the Chi square test was used to elucidate the *p* value. Cox-regression analysis was used to study the strength of the association between the baseline cancer status and all-cause mortality. To overcome the influence of competing risk of death, transplant and incident cancer, data were censored at the first occurring event in the regression models [11]. The Kaplan–Meier survival curve was also used to illustrate the cumulative survival between the groups. A competing risk analysis was also conducted using ‘cmprsk’ package of the R software [12]. The *p* value for the competing risk model was generated using modified *X*^2^ test (Gray, 1988) [13]. The rest of the analysis was performed using SPSS (version 22), licenced to the University of Manchester. The study complies with the declaration of Helsinki and local ethical approval has been obtained from the Research and Innovation department, Northern Care Alliance NHS group (Ref: S19HRANA09).

## Results

**Fig. 2:**
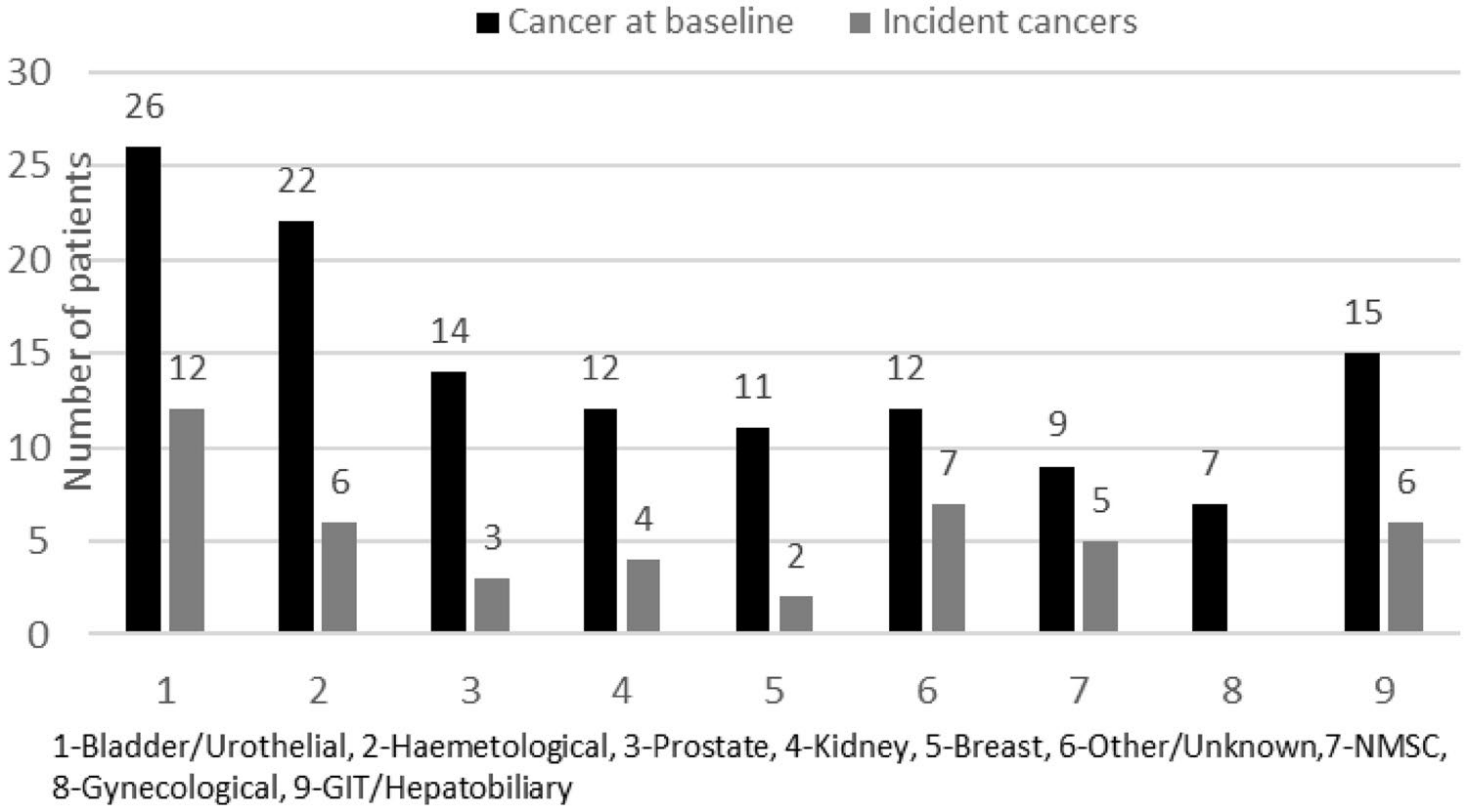
Cancer site distribution

10.1% (128/1271) of this ESRD cohort had a history of cancer at baseline with an annual incident rate of 1.3% throughout the study duration. The cancer site distribution is illustrated (number of patients in each cancer type) in Fig. 2. Urological cancers were the leading site for both prevalent and incident cancers, followed by haematological cancers.

At baseline, the median age of the cohort was 62 years, with patients in the cancer group being older (70 versus 60 years, *p* value < 0.001). There was a predominance of males (63%) and Caucasians (79%) in our cohort. The majority (69%) of the cohort were receiving haemodialysis. 47% of the cohort were diabetic with nearly 90% having a history of hypertension.

The groups were similar for most baseline comorbidities and cardiovascular event history apart from a higher prevalence of peripheral vascular disease noted in the cancer group (26% versus 18%, *p* value 0.02). The prevalence of chronic hepatitis infection (B&C) was 0.31% (4/1271). The biochemical parameters were similar in the two groups. Once propensity score matched for age, gender and ethnicity, the two groups were similar in all the baseline characteristics. Over a median follow-up of 28 months, there were more deaths noted in the cancer group (49.6% versus 39.6%, *p* value 0.02) although this difference was not observed in the matched sample. A significantly smaller percentage of patients in the cancer group received transplants compared to the no cancer group both in the total and matched sample (6% versus 28%, *p* value < 0.001) (Table 1).

**Table 1:** Baseline characteristics between patients with and without cancer in the total and the matched sample

Variable	Cancer (119)	No cancer (1152)	*p* value	Cancer (119)	No cancer (238)	*p* value
Age	70 (62.6–75)	60 (48–72)	**0.000**	70 (62.6–75)	69.6 (62–75.5)	0.84
Male	79 (66.4%)	721 (62.3%)	0.42	79 (66.4%)	170 (71.4%)	0.34
Ethnicity (Caucasian)	107 (89.9%)	893 (77.5%)	**0.002**	107 (89.9%)	216 (90.7%)	0.79
BMI	28.1 (24.3–32)	26.8 (23.4–31.1)	0.14	28.1 (24.3–32)	27 (23.4–31.4)	0.43
Systolic BP	140 (124–157)	143 (128–160)	0.19	140 (124–157)	143 (128–159)	0.35
Diastolic BP	73 (64–86)	78 (68–89)	**0.01**	73 (64–86)	73 (65–82)	0.99
Smoking	64 (53.8%)	519 (45%)	0.07	64 (53.8%)	134 (56.3%)	0.65
Alcohol	102 (85.7%)	890 (77.3%)	0.76	102 (85.7%)	185 (77.7%)	0.71
Modality (HD)	81 (68%)	792 (68.8%)	0.88	81 (68%)	172 (72.3%)	0.41
Hypertension	105 (88.2%)	1016 (88.2%)	0.99	105 (88.2%)	208 (87.4%)	0.82
Diabetes mellitus	53 (44.5%)	539 (46.7%)	0.64	53 (44.5%)	119 (50%)	0.33
Hypercholesterolemia	33 (27.7%)	302 (26.2%)	0.72	33 (27.7%)	71 (29.8%)	0.68
IHD	36 (30.5%)	327 (28.4%)	0.67	36 (30.5%)	75 (31.5%)	0.81
MI	13 (10.9%)	155 (9.9%)	0.44	13 (10.9%)	40 (16.8%)	0.14
CCF	37 (31%)	381 (33%)	0.66	37 (31%)	93 (39.1%)	0.14
PVD	31 (26%)	205 (17.8%)	**0.03**	31 (26%)	50 (21%)	0.28
CVA	6 (5.04%)	92 (%)	0.25	6 (5.04%)	25 (12.2%)	0.08
COPD	19 (15.9%)	180 (15.6%)	0.92	19 (15.9%)	50 (21%)	0.26
CLD	10 (8.4%)	100 (8.7%)	0.92	10 (8.4%)	23 (9.7%)	0.69
Chronic hepatitis (B&C)	0/119	4/1152	–	0/119	1/238	–
Dialysis vintage^a^	37.5 (20.9–51.9)	29.6 (11.9–54.1)	0.26	37.5 (20.9–51.9)	23.9 (11–44.4)	**0.04**
RAS blocker	61 (51.3%)	620 (53.8%)	0.59	61 (51.3%)	119 (50%)	0.82
Statin	81 (68%)	697 (60.5%)	0.11	81 (68%)	147 (61.7%)	0.24
HB (g/L)	102 (89–110)	101(89–112)	0.79	102 (89–110)	101 (89–112)	0.80
Albumin (g/L)	37 (33–39)	37 (32–40)	0.78	37 (33–39)	36 (32–39)	0.62
CRP (mg/L)	18.5 (5–43)	11(5–32)	**0.03**	18.5 (5–43)	12.5 (5–39)	0.26
Calcium (mmol/L)	2.3 (2.2–2.4)	2.33(2.2–2.4)	0.82	2.3 (2.2–2.4)	2.3(2.2–2.4)	0.99
Phosphate (mmol/L)	1.6 (1.29–1.97)	1.5 (1.2–1.9)	0.24	1.6 (1.29–1.97)	1.5 (1.2–1.85)	0.15
Ferritin (ug/L)	452 (262–683)	395 (218–670)	0.08	452 (262–683)	427 (237–703)	0.35
Follow up (months)	27 (17–47)	29 (16.7–47)	0.94	27 (17–47)	29.6 (17–48)	0.68
Death	60 (49.6%)	456 (39.6%)	**0.02**	60 (49.6%)	124 (52.1%)	0.77
Transplant	7 (5.9%)	321 (27.9%)	**0.000**	7 (5.9%)	38 (15.9%)	**0.007**

Statistically significant *p*-values are displayed in bold (i.e. *p* < 0.05)

Continuous variables are expressed as median (interquartile range) and *p* value by Mann–Whitney *U* test. Categorical variables are expressed as number (%) and *p* value by Chi Square test

*BMI* body mass index, *BP* blood pressure (mm of Hg), *DM* diabetes mellitus, *IHD* ischemic heart disease, *MI* myocardial infarction, *CCF* congestive cardiac failure, *CVA* cerebrovascular accident, *PVD* peripheral vascular disease, *COPD* chronic obstructive pulmonary disease, *CLD* chronic liver disease, *RAS* renin-angiotensin system, *HD* haemodialysis, *Hb* haemoglobin, *CRP* c-reactive protein

^a^Dialysis vintage was calculated for 455 patients on dialysis before the date of recruitment

**Fig. 3:**
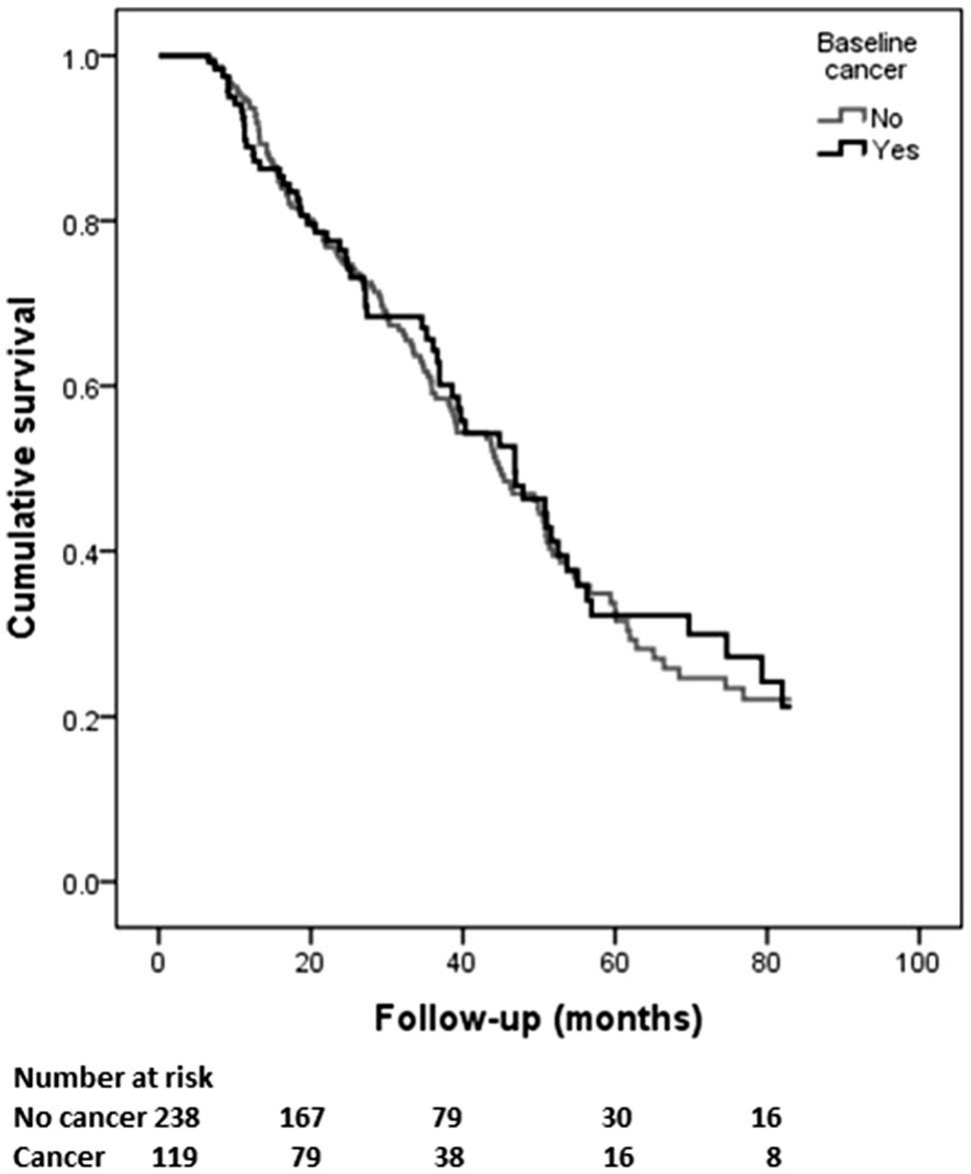
Kaplan–Meier chart comparing baseline cancer status with all-cause mortality in the matched sample

In a univariable Cox-regression model, a cancer history at baseline was found not to be associated with all-cause mortality in either the total or in the matched sample (total sample, HR 1.28; 95% CI 0.97–1.67; *p* = 0.07). Older age, diabetes, and the presence of cardiovascular events at baseline proved to be the risk factors associated with mortality in this model (Table 2). The Kaplan–Meier chart demonstrated similar observation with no difference noted in the cumulative survival in the presence of cancer (matched sample, Log-Rank *p* value 0.852) (Fig. 3).

**Table 2:** Univariable cox-regression model

Variable	Total sample		Matched sample	
	HR (95% CI)	*p* value	HR (95% CI)	*p* value
Cancer	1.28 (0.97–1.67)	0.08	0.97 (0.71–1.32)	0.85
Age	1.04 (1.03–1.05)	**0.000**	1.04 (1.02–1.06)	**0.000**
Male gender	1.1 (0.92–1.32)	0.286	1.18 (0.84–1.7)	0.35
Caucasian	1.39 (1.11–1.7)	**0.004**	0.98 (0.58–1.6)	0.94
Smoking	1.32 (1.11–1.57)	**0.001**	1.1 (0.82–1.48)	0.52
Diabetes	1.56 (1.31–1.86)	**0.000**	0.99 (0.75–1.3)	0.98
Modality (HD)	1.04 (0.86–1.26)	0.68	1.02 (0.73–1.41)	0.93
IHD	1.58 (1.33–1.88)	**0.000**	1.48 (11–1.98)	**0.008**
MI	1.73 (1.41–2.14)	**0.000**	1.44 (1.01–2.0)	**0.04**
CCF	1.86 (1.56–2.2)	**0.000**	1.52 (1.13–2.02)	**0.005**
PVD	1.43 (1.17–1.74)	**0.000**	1.27 (0.93–1.75)	0.14
CVA	1.60 (1.23–2.08)	**0.000**	1.56 (0.98–2.46)	0.06

Statistically significant *p*-values are displayed in bold (i.e. *p* < 0.05)

*IHD* ischemic heart disease, *MI* myocardial infarction, *CCF* congestive cardiac failure, *CVA* cerebrovascular accident, *PVD* peripheral vascular disease, *HD* haemodialysis

**Fig. 4:**
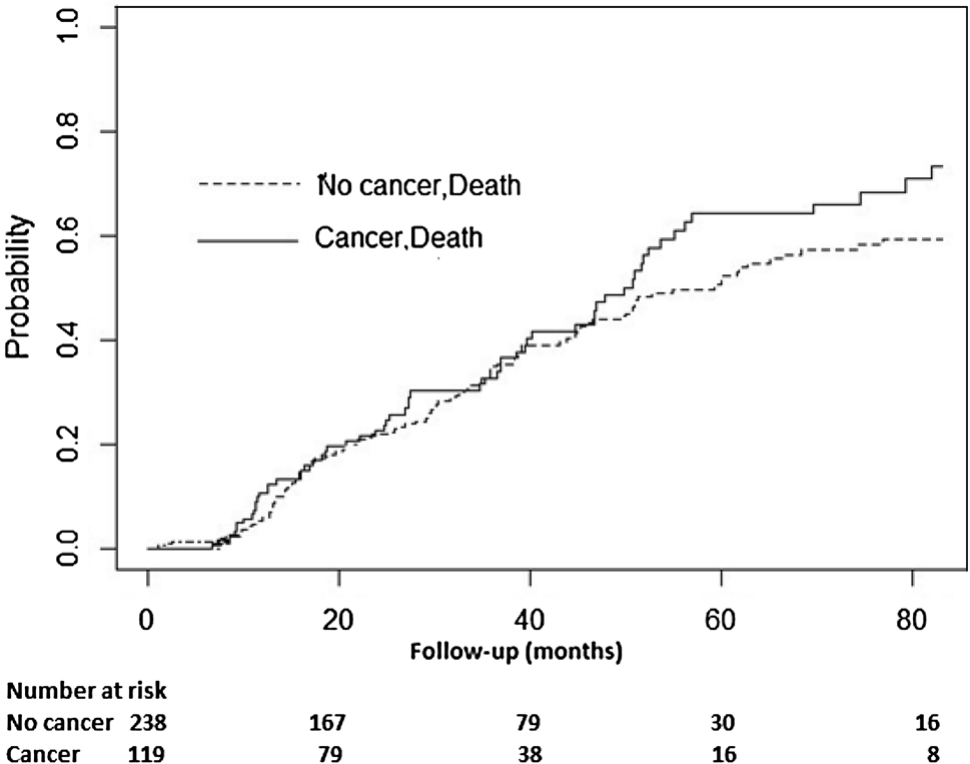
Cumulative probability of death between the groups in competing risk model in the matched sample

In a competing risk model including death, transplant and incident cancer as endpoints, a higher probability of death at 5 years was noted in the cancer group however the difference was not statistically significant (64% versus 51%, *p* value 0.16). The patients in the no cancer group had a significantly higher probability of having received a transplant at 5 years (18% versus 6%, *p* value 0.01) (Table 3 and Fig. 4).

**Table 3:** Cumulative incidence probability for death and transplant between the groups (cancer vs. non-cancer) in the matched sample

Months	Status (number at risk)	Death	Transplant
20	Cancer (79)	0.19	0.02
	No cancer (167)	0.18	0.05
40	Cancer (38)	0.40	0.07
	No cancer (79)	0.38	0.13
60	Cancer (16)	0.64	0.07
	No cancer (30)	0.51	0.18
	*p* value	0.16	0.01

## Discussion

Our study has given an overview of the distribution of various cancers and their association with all-cause mortality in a representative UK ESRD population receiving dialysis. The cancer prevalence rate of 10.1% and incident rate 1.3% are similar to observations in other dialysis cohorts [14, 15]. The prevalence of all cancer including NMSC in the UK general population in December 2015 was calculated to range between 2.4 and 3.9% across the regions (Public Health England data) and the incidence rate for all cancers in 2015 was 0.83% (Cancer Research UK). (https://www.cancerresearchuk.org/health-professional/cancer-statistics/incidence).

Urological (kidney, prostate, bladder and urinary tract) cancers followed by haematological cancers were the leading cancer distribution sites in our cohort which is in agreement with earlier reports [16]. The prevalence and incidence of hepatocellular carcinoma was low in our cohort compared to observations in East Asian populations which is likely due to a lower prevalence of viral hepatitis (0.31%), due to effective screening and immunisation practices [17, 18]. Patients with NMSC were included in the no cancer group for the comparative analysis as the 10-year mortality rate of patients with NMSC is reported to be the same as the general population [19].

The patients in the cancer group had a significantly higher median age at baseline compared to those without cancer. Association of old age with cancer is well documented [20]. A higher percentage of deaths was observed in the cancer group in the total sample, a likely reflection of the higher age in this group as this difference was not observed in the age matched sample. A significantly lesser proportion of patients in the cancer group received a kidney transplant, which may indicate a mindful risk-based approach in the transplant work-up [21].

In our study, a history of cancer did not prove to be an independent risk factor for all-cause mortality. The association of cancer with all-cause mortality in the ESRD population receiving dialysis has been variably reported. The mortality risk due to cancer in dialysis patients was noted to be similar to the general population in a multicentre study in Hong Kong by Cheung et al. [15], but few other studies have shown such an increased risk [22, 23]. The Dialysis Outcomes and Practice Patterns Study (DOPPS) in 2003 showed cancer as an independent risk factor to be associated with all-cause mortality (HR: 1.28, *p* < 0.001) [24]. In the ERA-EDTA registry study, among the non-cardiovascular causes of death in dialysis patients, infection-related mortality was increased by 82-fold while that of cancer-related mortality was 2.9 times higher than the general population [25]. In another large Japanese study comparing the mortality risk between the general population and dialysis patients, the all-cause mortality was found to be sevenfold higher in dialysis patients than the general population in the 60–74-year age group, which was mainly attributed to cardiovascular events and infection-related death (36 fold higher risk) while malignancies contributed to only a twofold higher risk [26].

The lack of association between the cancer status and all-cause mortality in our cohort may reflect a combination of increased mortality due to cardiovascular events and a careful choice in the provision of renal replacement therapy (RRT) options for ESRD patients. The difference observed in the uptake of kidney transplantation between the groups also strengthens this hypothesis.

Our study is limited by the single centre observational nature of the study methodology. Also, the lack of cause of death data limited our ability to identify the cancer-specific mortality risk. Our data was not able to capture the stage or treatment status of cancer, which might have had an influence on the results. In addition, we cannot exclude the possibility of a hidden selection bias in sampling, as cancer patients with limited expected survival may not choose to start RRT when ESRD supervenes.

In conclusion, there is an increased prevalence and incidence of cancer in ESRD patients receiving dialysis, with urological cancers being the leading cancer site, as might be expected by anatomical relationships. Baseline cancer history did not prove to be an independent risk factor for all-cause mortality in our multi-morbid dialysis population. There is a need for a careful case-by-case based approach in the provision of RRT options, including transplantation, in ESRD patients with cancer.

## Data Availability

The datasets used and/or analysed during the current study are available from the corresponding author on reasonable request.
